# Cheating 2.0: A reprofiling of the 10 most wanted test cheaters in the digital age

**DOI:** 10.1111/medu.15682

**Published:** 2025-05-20

**Authors:** Alison M. Sturrock, Gil Myers, Eliot L. Rees

**Affiliations:** ^1^ University College London School of Life and Medical Sciences London UK; ^2^ St George's University of London Institute for Medical & Biomedical Education, Cranmer Terrace London UK

## Abstract

In the last issue of Medical Education Unleashed, Royal et al (2016) introduced the “Ten Most Wanted” test cheaters. Almost a decade later, significant changes in assessment format and delivery have altered the landscape of academic dishonesty. This paper revisits the original 10 cheaters, updates their statuses and identifies new contenders.

Cheating remains a pervasive issue in medical education, exacerbated by the shift to online assessments during the COVID‐19 pandemic. In medical education, cheating threatens assessment validity, patient safety and institutional reputation, and may correlate with future unprofessional behaviour. The motivations behind cheating are multifaceted. We use the ‘fraud triangle’ of motivation, opportunity and rationalisation as a framework to conceptualise some of these. This paper profiles the evolution of cheating methods, highlighting new digital‐age strategies. An approach which combines authentic assessments, technological integration and promotion of academic integrity is essential to combat this enduring issue.

## Abstract 2

Here we go again … as digital technologies continue to expand and pressure on students remain high, it's time for a new look at cheating behaviours #MedEducUnleashed

## 1 INTRODUCTION

In the last issue of Medical Education Unleashed, Royal and colleagues introduced us to the “Ten Most Wanted” test cheaters in medical education.^1^ Almost a decade has passed, bringing significant changes in how assessments are delivered. With the advent of computer‐based testing, a global pandemic, and the emergence of generative AI, some notorious cheaters have been defeated or made irrelevant, whereas crafty new contenders have surfaced. In this paper, we will revisit the original 10, catch up on those still at large, and unveil the new architects of deceit.

Cheating is a problem prevalent throughout higher education.^2^, ^3^, ^4^ A recent study revealed that 16% of students admitted to cheating within the past year, with only 5% being caught red‐handed.^5^ The shift in assessment formats during the COVID‐19 pandemic further exacerbated this problem. Before the pandemic, 30% of students admitted to cheating; after the pandemic that number had nearly doubled to 55%. The most common reason for cheating was simply that ‘there was an opportunity to do so’.^6^

**FIGURE 1:**
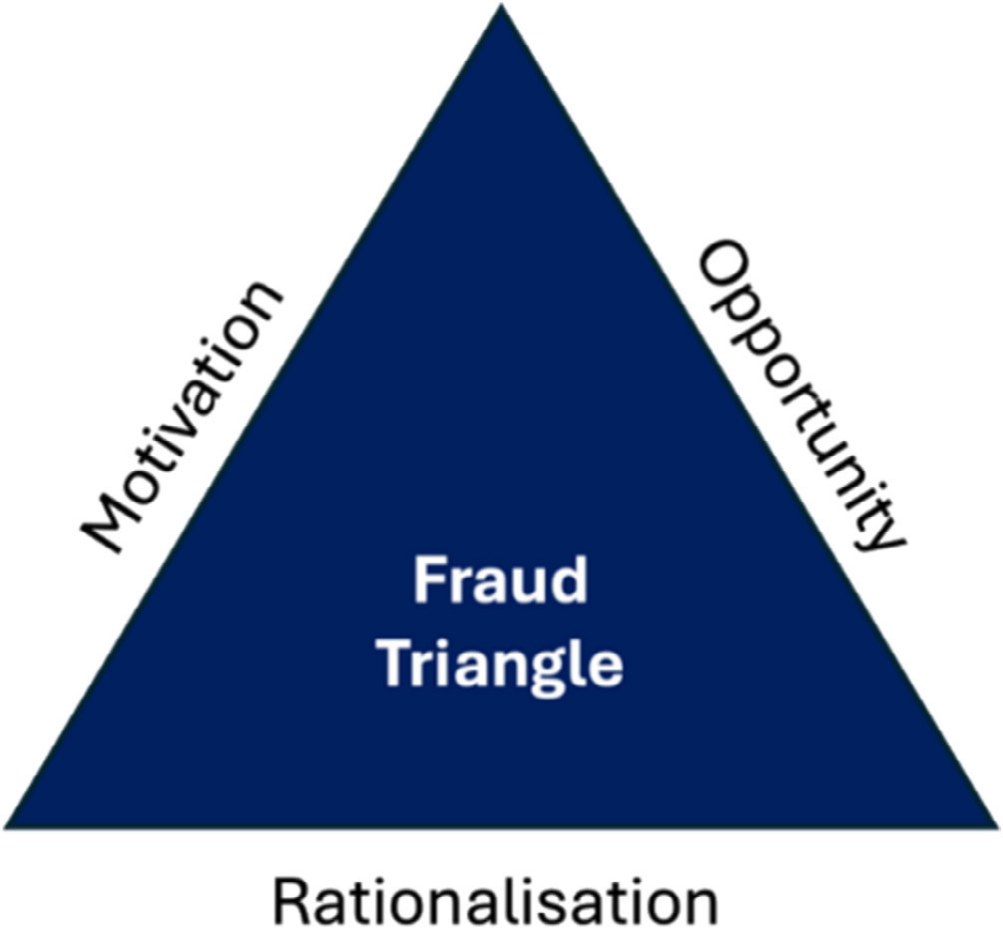
The fraud triangle. [Color figure can be viewed at wileyonlinelibrary.com]

The motivations behind cheating are complex and can be explained by the ‘fraud triangle’—a framework consisting of motivation, opportunity and rationalisation. Originally used to understand financial fraud, this model has recently been applied to higher education (Figure 1).^7^

Cheating thrives in environments where it is easy to pull off, rewards are great and risk of getting caught is low. Perhaps the most damning rationale is the perception that “everyone else is doing it,”^8^, ^9^ leaving honest students suffering unfairly for their integrity.

In medical education, cheating is more than just a breach of academic integrity; it threatens assessment validity, jeopardises patient safety,^4^ and tarnishes an institution's reputation. Even more concerning is the potential link between cheating in school and future unprofessional behaviour—a pipeline we would rather not build.^10^

## 2 THE 10 MOST WANTED CHEATERS

In our updated list of the 10 most wanted, some of the originals have been caught, many have evolved to remain at large and new classes of cheaters have emerged (Table 1).

**TABLE 1:** A sit rep on Royal et al’s 10 most wanted cheaters.

Original cheater		Cheater 2.0
**The smuggler** Brought cheating contraband into the exam hall	**➔**	**The amnesiac**
**The tourist** Had roaming eyes in the exam hall	**➔**	***Caught** by computer‐based assessments randomising items and response orders*
**The incontinent** Frequently visited the toilet. Not for biological reasons	**➔**	***Caught** by the introduction of stricter rules and restroom inspections*
**The impersonator** Took the assessment on someone else's behalf	**➔**	**The avatar**
**The hacker** Downloaded material and compromised security	**➔**	**The video editor**
**The storyteller** Shared exam experiences down generations of students	**➔**	**The content curator**
**The air traffic controller** Directed traffic to elicit websites and online material	**➔**	**The mastermind**
**The collaborator** Hosted group workers when the task requires one brain only	**➔**	**Team‐workers**
**The empathiser** Previous candidates providing current ones with exam materials	**➔**	***Caught** by large banks of assessment items and varying content of clinical skills assessments*
**Robin Hood** A current educator correcting failing students' scores	***➔***	***Caught** by raising awareness of the implications of failure to fail*.

### 2.1 The smuggler becomes the amnesiac

When online examinations occur at home, smugglers no longer need to carry their material anywhere. Now they just “forget” to remove helpful Post‐it notes, camouflaged diagrams and advantageous reminders hidden in plain sight around their screen.

### 2.2 The impersonator becomes the avatar

An online impersonator will find it easier to take on the identity of another student. Where there are security checks, a low‐resolution web camera and quickly waved, out‐of‐focus ID often passes the brief checks where the attention is on the correct name rather if the person in front resembles their photo ID taken when they were a fresh‐faced first year.

### 2.3 The storyteller becomes the content curator

Half‐remembered OSCE scenarios or clinical questions can be checked and organised into a fully fleshed‐out analysis. Cloud storage and group messages mean an exam paper can be collected, collated and circulated before the assessment window closes. Everyone contributes to a shared document^11^; the curator summarises and highlights to make the raw material easier to navigate.

### 2.4 Collaborators become team‐workers

Remote assessments increase the opportunity for collaboration with students chatting on message groups. When the assessment is proctored, there are safeguards to block these programmes, so cooperation needs to become planned rather than opportunistic. For example, a virtual desktop running the safe exam browser alongside the chat.

### 2.5 The hacker becomes the video editor

Assessment security has substantially increased, however, IT‐literate students are putting their skills to new use. When students are expected to submit recordings of their patient interactions there is the opportunity to edit the recording. They can give themselves a second attempt, edit out that part where the consultation got derailed, or just speed up the video to make sure everything was achieved within the time limit.^12^ Students may also rationalise this if they do not believe this is an authentic assessment.

### 2.6 The air traffic controller becomes the mastermind

Behind the scenes, there is someone who orchestrates a sophisticated network of students^13^ – maybe even educators – connected through encrypted WhatsApp groups. They meticulously gather exam material, sometimes paying or coercing (if you do not share the questions, you do not get the resources) insiders to leak this information. Once the resources are gathered, they distribute the answers to their network. This mastermind's operations are highly organised, making it challenging for faculties to catch them in the act.

Four new types of most wanted cheaters have emerged:

### 2.7 The tempted

Those that “wouldn't steal a car”^14^ may be tempted to check on their handheld device if they remembered something correctly. Philosophical debates can be had of whether it's cheating to use a resource to safely manage a patient. However, the less authentic the assessment becomes the more an average student will be able to rationalise the temptation to “cheat”.^15^, ^16^

### 2.8 The fabricator

Technology does not yet allow a student to fabricate a convincing video using AI, but ePortfolios can be faked,^17^ patients taken from online examples or clinical material scanned, photoshopped and edited. Forging a written signature requires sufficient penmanship but creating an email or ticking an electronic box is easily achieved with a high expectation of going unnoticed.

### 2.9 The entrepreneur

Social Media clips can drive up interest when the viewer agrees to “like, share and subscribe”. The (relative) altruism of helping peers can be monetised to gain influencer status (#medfluencer).^18^ Whilst many find these posts educational, inspiring and empowering they can be sold to reveal critical assessment content or cloned responses to recurrent assignments.

### 2.10 CheatGPT

Any student can, by inputting assessment questions into ChatGPT or alike, receive detailed answers and justifiable explanations, undermining the integrity of the assessment process. This misuse not only compromises the fairness of the exams but also poses significant risks to patient safety, as it may result in underqualified individuals advancing in their medical training.^4^

## 3 APPROACHES TO PREVENTION AND DETECTION OF CHEATING

To prevent and detect cheating, institutions should employ technological and psychological approaches to address the three domains of the fraud triangle.

### 3.1 Motivation: reducing pressure and temptation

Single‐moment‐in‐time high‐stakes exams (REF) increase the motivation to cheat; replacing these with frequent, low‐stakes assessments reduces student anxiety and makes cheating less appealing.^19^, ^20^ Offering mental health resources and academic support services address the stress‐related motivations to cheat, making honesty the easier path for the ‘tempted’ student.

Authentic, case‐based, open‐book assessments that mirror real‐world scenarios can also reduce the temptation to cheat; accessing outside material offers little advantage since these tests focus on critical thinking and problem‐solving skills.^21^ It is like trying to cheat in a cooking competition by bringing a recipe—if you cannot cook, it will not help!

Alternatively, tactics which get into the head of a potential cheater to demotivate them are impactful: a stern pre‐exam pep talk, warning that every student's *ClickMaps* ^22^ are monitored for anomalies or posters in the exam hall featuring a “rogues' gallery of cheaters”.

### 3.2 Opportunity: restricting the means to cheat

Preventing cheating is like playing a game of whack‐a‐mole, but with more tech and no hammer. In online assessments, AI‐driven proctoring systems act as digital hawks, watching for suspicious behaviour such as shifty eyes or illicit devices. Biometric verification ensures that the student taking the exam is genuine and not their overachieving cousin. This makes impersonation or utilising external help as difficult as finding that ‘needle in a haystack’.^23^

In computer‐based assessments, secure browser technology locks down the exam environment, making unauthorised resource access less likely. Randomising questions from a vast bank gives each student a unique test, turning collaboration into a game of “guess what I got” diminishing collaboration or content sharing.^23^ If that is not feasible, randomising questions, option lists and seating arrangements can still keep students on their metaphorical toes.

In any assessment, well‐trained proctors are the unsung heroes, vigilantly watching for signs of cheating from covert note‐passing to hidden communication devices. Encouraging proctors to challenge suspicious behaviour is crucial to deter others and keep the exam environment as fair as is possible.

### 3.3 Rationalisation: shaping ethical culture and awareness

Rationalisation happens when students convince themselves that cheating is okay. To tackle this, we must build a strong culture of academic integrity. Institutions can shout about the importance of honesty and the serious consequences of cheating via honour codes, ethics training and regular campaigns.^24^ Connecting academic dishonesty to real‐world disasters, like a doctor misdiagnosing a patient, should make students think twice before justifying their actions.

Educational campaigns should spotlight the importance of honesty, the long‐term benefits of genuine learning and the penalties for cheating. By making consequences crystal clear and consistently enforcing them, we refute the idea that cheating is a “victimless crime.”^25^

Encouraging students to report cheating through anonymous systems also helps combat the normalisation of dishonest behaviour. When students see that cheating is a no‐go among their peers,^26^ they are less likely to justify their own misconduct. Professional role modelling is crucial^27^; students often mimic their seniors' behaviour, whether it's good or bad.

## 4 CONCLUSION

Institutions, despite being armed with robust evidence, often exhibit a curious reluctance to act. Contemporary cheating may seem like a modern‐day Hydra, sprouting new heads with each technological advancement, however, it is, in fact, an age‐old nemesis that can be defeated with consistent practice.

Staying ahead of these test cheaters is achievable. It requires a multi‐faceted approach which blends authentic assessments (motivation), the integration of value‐added technology (opportunity) and the promotion of academic integrity (rationalisation). It is also imperative to remember that fostering good behaviour is as crucial as punishing the errant.

## AUTHORS' CONTRIBUTIONS

All authors meet the ICMJE criteria for authorship. All authors contributed equally to the conception, design, drafting and revising of this project. All authors have read and approved the final manuscript.

## ETHICS APPROVAL AND CONSENT TO PARTICIPATE

The authors have no ethical statement to declare.

## DETAILS OF ANY ACKNOWLEDGEMENTS

We would like to thank Professor I C McManus for his contribution to our discussions on cheating.

## DETAILS OF ANY POTENTIAL CONFLICT OF INTEREST

The authors have no conflict of interests to declare.

## DETAILS OF ANY ACKNOWLEDGEMENTS

We would like to thank Professor I C McManus for his contribution to our discussions on cheating.

## DETAILS OF ANY POTENTIAL CONFLICT OF INTEREST

The authors have no conflict of interests to declare.

## Data Availability

Data sharing not applicable to this article as no datasets were generated or analysed during the current study.
